# How much metagenome data is needed for protein structure prediction: The advantages of targeted approach from the ecological and evolutionary perspectives

**DOI:** 10.1002/imt2.9

**Published:** 2022-03-06

**Authors:** Pengshuo Yang, Kang Ning

**Affiliations:** ^1^ Key Laboratory of Molecular Biophysics of the Ministry of Education, Hubei Key Laboratory of Bioinformatics and Molecular‐Imaging, Department of Bioinformatics and Systems Biology Center of AI Biology, College of Life Science and Technology, Huazhong University of Science and Technology Wuhan Hubei China

**Keywords:** ecology, evolution, metagenome data, protein 3D structure modeling, targeted approach

## Abstract

It has been proven that three‐dimensional protein structures could be modeled by supplementing homologous sequences with metagenome sequences. Even though a large volume of metagenome data is utilized for such purposes, a significant proportion of proteins remain unsolved. In this review, we focus on identifying ecological and evolutionary patterns in metagenome data, decoding the complicated relationships of these patterns with protein structures, and investigating how these patterns can be effectively used to improve protein structure prediction. First, we proposed the metagenome utilization efficiency and marginal effect model to quantify the divergent distribution of homologous sequences for the protein family. Second, we proposed that the targeted approach effectively identifies homologous sequences from specified biomes compared with the untargeted approach's blind search. Finally, we determined the lower bound for metagenome data required for predicting all the protein structures in the Pfam database and showed that the present metagenome data is insufficient for this purpose. In summary, we discovered ecological and evolutionary patterns in the metagenome data that may be used to predict protein structures effectively. The targeted approach is promising in terms of effectively extracting homologous sequences and predicting protein structures using these patterns.

## Highlights

- Metagenome benefits for homologous sequence supplement for protein three‐dimensional (3D) structure prediction.
- Metagenome utilization efficiency shows a divergent distribution of proteins.
- Marginal effect model also quantifies this divergent distribution of proteins.
- For mining homologous sequences, the targeted approach outperforms the untargeted approach.
- Current metagenome data is not enough for modeling 3D structures for all proteins.

## Graphical abstract

For protein 3D structure prediction, we mine the data‐dependent ecological and evolutionary trends hidden in metagenome data. Based on this pattern, the targeted approach was presented to predict the protein 3D structure more effectively and accurately than the untargeted approach's blind search.

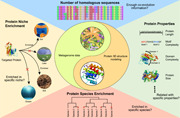

## INTRODUCTION

It has been proven feasible that protein three‐dimensional (3D) structures could be modeled with the supplement of metagenome sequences as homologous sequences. However, although a large amount of metagenome data is used for such purposes, a considerable number of proteins could still not be modeled. Such phenomenon has attracted our attention: is there any metagenome data‐dependent patterns behind, what are the intricate but potentially important properties about protein structures that lead to such patterns, and how to best utilize such properties for better protein structure prediction. More importantly, it was suspected that the reason behind this is tightly related to the ecological and evolutionary patterns of the metagenome sequence utilization based on different niches (i.e., biomes).

Here we focused on the divergent distribution of homologous sequences for protein families in the different metagenome and conducted a biome‐aware assessment for different performances of metagenome‐based protein 3D structure prediction methods. Firstly, to detect the divergent distribution of homologous sequences in the metagenome from different biomes, metagenome utilization efficiency is proposed, which is defined as the proportion of aligned homologous sequences in all metagenome sequences. The analysis of utilization efficiency on the ecological and evolutionary perspective shows a biome‐dependent homologous sequences distribution for a protein family. Secondly, as a model to illustrate the different potential of metagenome data from different biomes in supplementing the homologous sequences for protein structure modeling, the marginal effect model could also quantify this divergent distribution. Thirdly, constructed based on this pattern, the targeted approach could find enough homologous sequences from targeted biomes rather than the blind search used in the untargeted approach. The benchmark result shows that the targeted approach needs much fewer metagenome sequences and results in a more precise model compared to the untargeted approach. Finally, the lower bounds for metagenome data needed for protein structure prediction have been estimated and the results show that current metagenome data (roughly 1.48E12 metagenome sequences) is still far from enough for reliable protein structure prediction (roughly 7.12E12 metagenome sequences). And the targeted approach would partially overcome this challenge by lowering this bound to around 4.32E12 metagenome sequences due to higher utilization efficiency.

Collectively, our assessment of the utilization efficiency and the marginal effect has revealed strong ecological and evolutionary patterns behind the metagenome data for effective protein structure prediction. Utilizing these patterns, the targeted approach is promising in reliably excavating homologous sequences and predicting protein structures.

## PROTEIN 3D STRUCTURE PREDICTION

It has always been fascinating how proteins, in their native structures, could function in a species [1, 2, 3], leading to the central topic of how protein structure is associated with protein functions. Modeling the 3D structure of proteins is a computer method for better understanding this important subject [4, 5]. A major challenge, however, is that the number of ways a protein could theoretically fold before settling into its final 3D structure is astronomical [6, 7, 8, 9]. However, proteins fold spontaneously in nature, some within milliseconds—a dichotomy sometimes referred to as Levinthal's paradox [10, 11]. These findings may allow for more accurate drug development efforts, complementing existing experimental approaches to uncover potential therapies more quickly [12, 13]. Furthermore, some published tools offer the ability to investigate the hundreds of millions of proteins for which we presently lack models—a big territory of undiscovered biology [14, 15, 16]. There may be proteins with novel and intriguing functions among the unsolved proteins, much as a telescope allows us to view deeper into the undiscovered cosmos [17, 18, 19].

Determination of protein 3D structure is usually conducted by wet‐lab experiments [20, 21, 22]. X‐ray crystallography, nuclear magnetic resonance spectroscopy, and electron microscopy are some of the technologies now utilized to identify the structure of a protein [23, 24, 25]. To develop the final atomic model, the scientist employs several bits of information in each of these methods [26, 27]. However, because experimental approaches are often slow and arduous, thus for many proteins, computational methods are usually employed to determine, or more precisely predict, the protein 3D structures, with varying resolutions [26, 28, 29].

## TEMPLATE‐FREE PROTEIN 3D STRUCTURE PREDICTION

Protein 3D structures are usually predicted through two approaches: template‐based and template‐free [5, 30, 31]. Template‐based protein structure prediction (also known as homology or comparative modeling) employs knowledge of solved structures to model the native or true fold of a protein sequence [32, 33, 34]. Template‐based protein structure prediction has long been thought to have tremendous potential for producing atomically precise models that are close to the native conformation [35, 36]. However, because the template‐based method is strongly reliant on an existing solved structure, it can only be used for a restricted number of proteins [37, 38].

Template‐free methods are currently big‐data‐driven methods that are based on homologous protein sequences and multiple sequence alignment (MSA) to predict protein structures without any known template [39, 40, 41]. The template‐free method relies on a large amount of high‐quality homologous sequences to make accurate predictions [14, 42, 43]. Currently, several representative template‐free methods are widely used for protein 3D structure prediction, including Rosetta [42], Iterative Threading ASSEmbly Refinement (I‐TASSER) [5], and AlphaFold [44]. Rosetta [42] is a long‐standing software system for predicting protein structure well‐known for its versatile functionalities and diverse applications [45, 46, 47]. I‐TASSER is also a long‐standing software system for protein structure prediction [5]. Empowered by deep learning methods, I‐TASSER performs well in the field of template‐free protein structure prediction [48, 49]. Most importantly, recent AlphaFold predicted extremely high‐accuracy structures for 87 out of 92 domains in the CASP14, outperforming other methods [44, 50, 51]. All these template‐free tools' achievements rely substantially on homologous sequences, implying that homologous sequences are crucial for template‐free protein 3D structure prediction [16, 52, 53].

In summary, template‐free methods are currently commonplace in protein structure prediction, and several template‐free methods are utilized to predict huge batches of proteins. On the one hand, deep learning techniques have made it possible for template‐free methods to predict protein structures at unprecedented speed and accuracy. On the other hand, template‐free methods are usually dependent on homologous sequences of the proteins, which should be plentiful and diverse within themselves. And these requirements for homologous sequences have resulted in the formation of a huddle for template‐free protein 3D structure prediction.

## CURRENT PROBLEMS FOR TEMPLATE‐FREE PROTEIN 3D STRUCTURE PREDICTION

**Figure 1:**
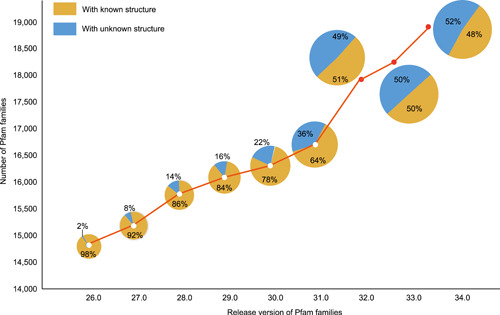
The number of Pfam families under release version changes up till Pfam version 34.0. The curve illustrates the number of Pfam families ranged by the release version. The pie charts attached to the corresponding release version reflect the proportion of Pfam families with known and unknown structures

Everything has two or multiple sides, protein 3D structure prediction is not an exception [54, 55, 56]. On one side, current methods, particularly AlphaFold, have already enabled the accurate structure prediction across more than 365,198 proteins for 21 species, resulting in an average coverage of 80.45% for all the proteins in reference proteome, including nearly all proteins (coverage over 99%) in six species [57]. On the other side, many proteins, including those in the Pfam database, have unknown 3D structures, and this number is also soaring rapidly [58, 59, 60]. In Pfam 26.0, only 2% of proteins lack structural information, but in Pfam 34.0, more than 50% of proteins do not have structural information (Figure 1). This phenome would be due to the contradiction between the advanced sequencing technology to find out more novel proteins and the limited development of wet experiment technology or the limited homologous sequences to identify their 3D structures [38, 61, 62].

These facts have resulted in an ostensibly but sensible trend: While the structures of more and more proteins are being predicted with increasing precision, there are also more and more proteins emerged that have no structure. This is rational because more and more species have been sequenced, leading to more and more proteins. As most of these are novel proteins, their protein 3D structures are not readily available. Faced with the increasing number of novel proteins, there is an urgent need to effectively find all available homologous sequences for template‐free protein 3D structure prediction.

## PREDICTION OF PROTEIN 3D STRUCTURE USING METAGENOME SEQUENCES

One possible solution for the prediction of no known protein structure problem is by means of using metagenome sequence data to supplement the homologous sequence [63, 64, 65, 66]: As a big reservoir of functional genes, metagenome could supply a considerable amount of homologous sequences for proteins [67, 68, 69]. Combined with more homologous information and an advanced template‐free prediction pipeline, many proteins with unsolved structures would be modeled with reliable structures. However, regardless of the protein structure prediction technique used, “more sequences lead to more protein structure predictions” is not true in most circumstances [63, 64]. Using over two billion proteins from different metagenome samples (mostly from the Gut microbiome), Baker et al. [63] could predict protein structures for 614 proteins with unknown structures in the Pfam database. While by only utilizing 97 million proteins from Ocean metagenome data, Zhang et al. [64] could predict protein structures for 27 proteins that cannot be solved in Baker et al.'s work. Most recently, by using 4.25 billion microbiome sequences from four biomes (Gut, Lake, Soil, Fermentor), Yang et al. [70] could predict protein structures for 1044 proteins in the Pfam database. All these findings suggested that metagenome sequences could supplement homologous sequences for protein 3D structure prediction and that this supplement has a significant biome‐related divergence.

**Figure 2:**
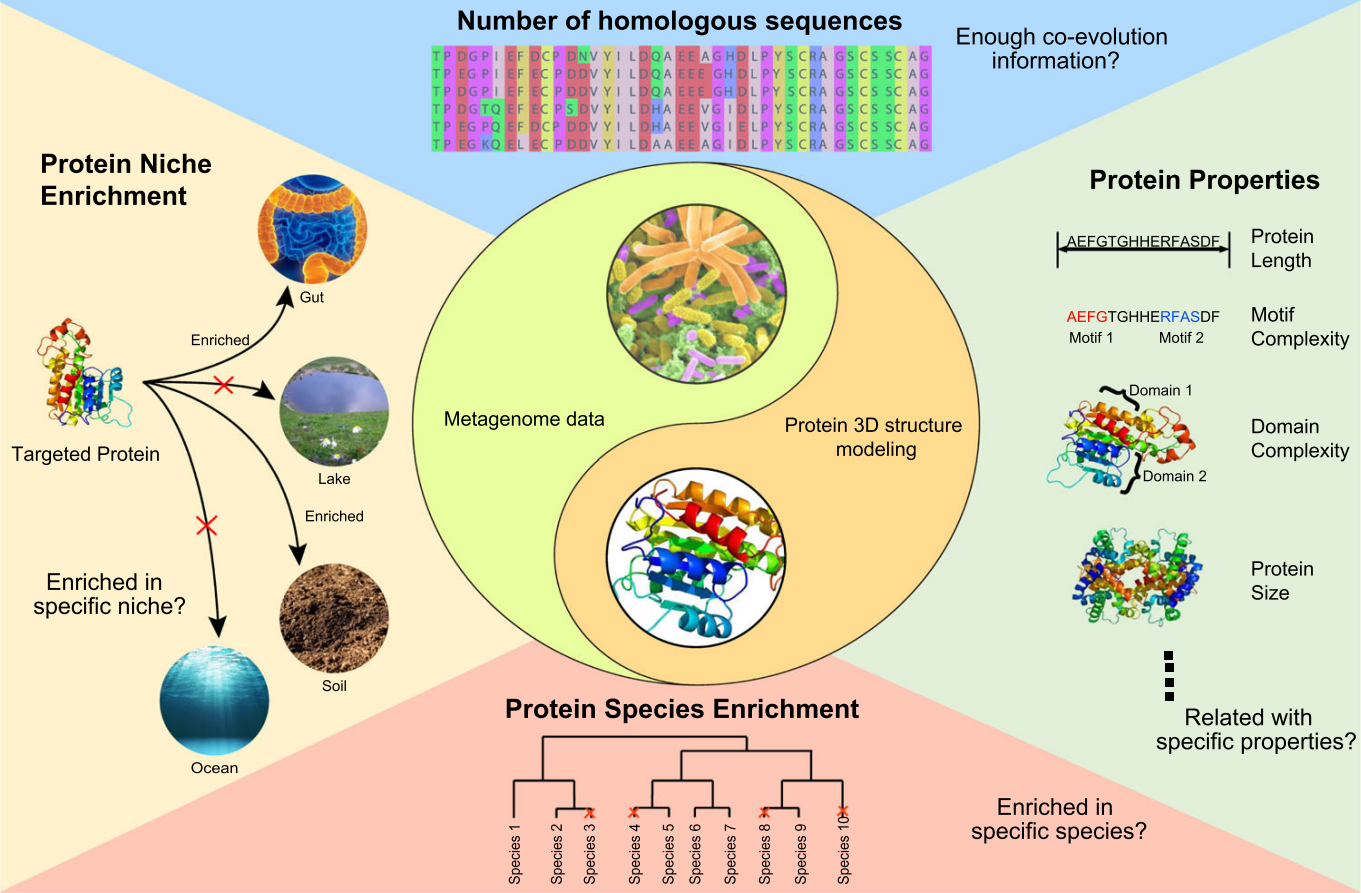
Examining the data‐dependent ecological and evolutionary patterns behind the metagenome data from multiple aspects. To examine the correlation between metagenome and proteins in Pfam, evolutionary patterns, including the number of homologous sequences and protein properties, would be investigated. Moreover, the ecological patterns, including the enrichment patterns of source species and metagenome niche, would also be investigated

Thus, two questions are obvious: what means we can utilize metagenome data for protein structure prediction? And how much metagenome data is needed for protein structure prediction? For both questions, the key objectives lay ahead: *effective homologous sequence supplement*. It would be vital to investigate what factors have affected the process of prediction of protein structure from metagenome data and find ways to best utilize these metagenome data properties to discover protein 3D structures for more proteins. To answer these critical questions, we have examined the data‐dependent patterns behind the metagenome data, from the ecological and evolutionary perspectives aspects (Figure 2). Using the successfully modeled proteins supplemented by metagenome data with unsolved structures in the Pfam database as a benchmark data set, we would investigate their evolutionary patterns (number of homologous sequences; protein function) and the ecological patterns (the enrichment patterns of source species and metagenome niche).

## ESTIMATION OF THE METAGENOME UTILIZATION EFFICIENCY

**Figure 3:**
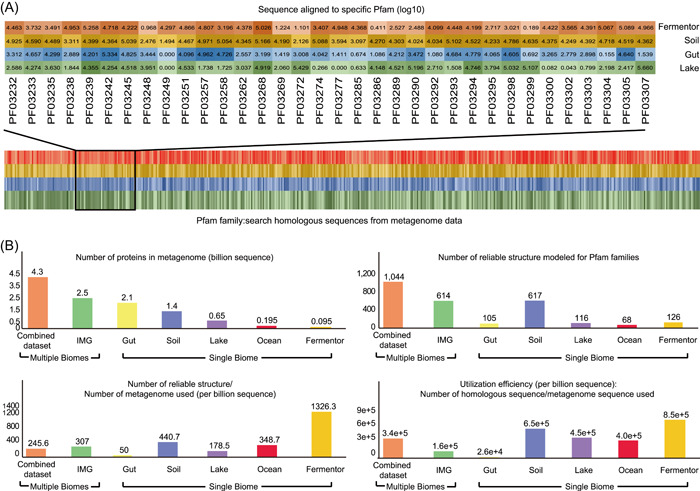
Metagenome sequence utilization efficiency evaluation. (A) Supplemented by the metagenome data set from different biomes, the homologous sequences were aligned to all the Pfam families, exemplified by metagenome from four biomes. Different color means their source biome and the shade of the color represents the number of metagenome sequences aligned to the corresponding Pfam families (the darker, more sequences aligned). (B) After homologous sequences aligned, the number of Pfam families predicted with reliable structures was calculated. Averagely, after using metagenome sequences (billion sequences), the number of homologous sequences aligned, and reliable structure modeled were calculated. Then, the metagenome sequence utilization efficiency was evaluated by calculating the proportion of the number of Pfam families in the number of metagenome sequences and the proportion of the number of supplemented homologous sequences in all the metagenome sequences

With the explosive growth of microbiome data, searching homologous sequences in metagenome for protein requires a huge search space and a significant amount of time [71, 72, 73]. As a result, metagenome utilization efficiency is the key to the successful prediction of protein structure from metagenome data. “Metagenome utilization efficiency” is defined as the proportion of homologous sequences that could be used for MSA supplement, among all sequences examined. Apparently, a greater metagenome utilization value showed that employing metagenome data for protein structure prediction was more successful. It was also clear how to boost metagenome utilization: either increase the number of homologous sequences that might be utilized for MSA supplementation or limit the protein sequence search space. In this review, the effectiveness of using metagenomes from diverse biomes to complement homologous sequences was assessed (Figure 3).

First, the homologous sequences of all the Pfam families are searched against metagenome from different biomes to evaluate the utilization efficiency (Figure 3A), which has been utilized to model the reliable structures for Pfam families (Gut, Soil, Lake, Fermentor and combined four data set [70], multiple biomes from IMG database [63] and Ocean [64]). Then using per billion metagenome sequences, the number of reliable proteins structures modeled and the number of supplemented homologous sequences was calculated (Figure 3B). For combined data set from four biomes (Soil, Lake, Fermentor, and Gut), highly reliable folds were modeled for 1044 Pfam families supplemented by 4.25 billion metagenome sequences, accounting for 12.00% of 8700 Pfam families with unsolved structures, higher than those in previous works [63, 64] and one of the four biomes [70]. However, utilizing the combined data set has not been demonstrated to be more efficient. Using the soil biome as the representation of a single biome, 9.1e+5 homologous sequences were detected, and the utilization efficiency would be calculated as 6.5e+5 per billion metagenome sequences (9.1e+5 homologous sequences/1.4 billion of sequencing data used). However, for the combined data set, though 14.6e+5 homologous sequences were detected, the utilization efficiency was only 3.4e+5 per billion metagenome sequence (14.6e+5 homologous sequences/4.3 billion of sequencing data used), much lower than those based on a single biome. The same result would be also deduced when using the IMG database, which includes multiple biomes, than single biomes (Figure 3B). This utilization efficiency analysis shows that if we have targeted the source biomes for the specific protein families, then protein sequences from single biomes considered in this study are significantly more efficiently used than using the data from different biomes.

Taken together, the efficiency of metagenome utilization is extremely biome‐dependent on a global view. Under particular environmental stresses in a given niche (i.e., biome), some genes may evolve so that the host species could better adapt to the environment, according to the ecological perspective on gene or protein evolution. Point mutations or gene structural variations might develop during this process and accumulate throughout generations of species [70, 74, 75]. As a result, we could frequently find a collection of homologous sequences for one protein under one biome. These proteins would aid the host's survival. Hence, choosing the proper biome for a single protein will greatly increase metagenome utilization and give a hint to derive the protein function for its host.

## MARGINAL EFFECT FOR PROTEIN STRUCTURE PREDICTION

**Figure 4:**
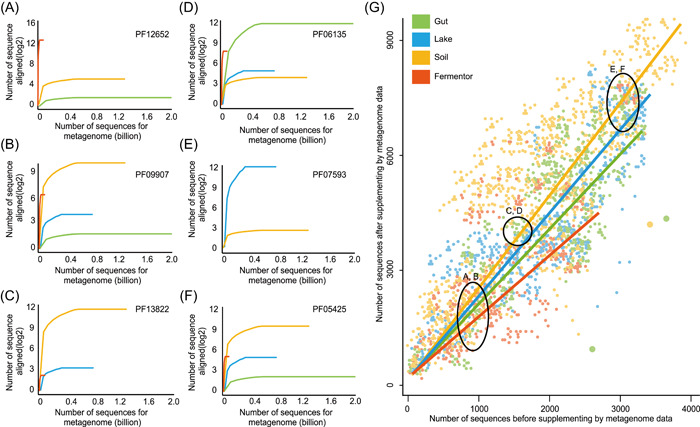
Marginal effects evaluation. Based on the data in reference [70], the marginal effects of the four biomes (Gut, Lake, Soil, Fermentor) on all the 8700 unknown Pfam families (version 32.0) were evaluated, described in reference [70]. The background is an ontology structure that contains the protein families and their relationships, while different colors indicated the high marginal effect values for that protein family by a certain biome. The marginal effect values are also annotated beside several proteins of interest. The data show that the contributions of different biomes to a specific Pfam can be drastically different, as reflected by their marginal values

The term “marginal effect” generally refers to a data set's ability to solve a certain problem [76, 77, 78]. In the context of protein structure prediction, the “marginal effect” ME (*B*_i_, *P*_j_) is defined as the potential of metagenome data from a given biome *B*_i_ in supplementing homologous sequences for a certain protein *P*_j_. The higher marginal effect usually indicated higher utilization efficiency if we use metagenome data from biome *B*_i_ for supplementing homologous sequences for protein *P*_j_. Exemplified by PF12652, estimated by marginal effect model, up to 6218 homologous sequences could be aligned by the Fermentor biome but only 24 homologous sequences could be aligned from the Soil biome. The actual alignment of homologous sequences from the metagenome in the Fermentor and Soil biomes may corroborate this marginal effect result (Figure 4A): For PF12652, 4125 homologous sequences could be aligned from the Fermentor biome, and 18 homologous sequences could be aligned from the Soil biome. Hence, for PF12652, the metagenome from the Fermentor biome could have a higher potential to supplement the homologous sequences than the Soil biome.

We evaluated marginal effects on the four biomes (Gut, Soil, Lake, Fermentor) [70] to supplement the homologous sequences for all the 8700 unsolved Pfam families, with results showing that big biomes such as Soil, which contains many metagenome samples and sequences, usually have high marginal effect values for the majority of proteins, but this is not a “winner takes all” pattern. For many proteins, small biomes like Fermentor could also have high marginal effect values (Figure 4). From an evolutionary standpoint, metagenome sequences in various biomes might have distinct evolutionary information (i.e., homologous sequence) for individual proteins.

## OTHER FACTORS THAT MIGHT IMPACT THE SUCCESS OF PROTEIN STRUCTURE PREDICTION USING METAGENOME DATA

First, from the evolutionary perspective, the approach of protein structure prediction using metagenome data were characterized as a strategy that “exhausts all attempts in discovering close sequences.” Hence, variables affecting the quality of MSA would impact the success of protein structure prediction using metagenome data. As an important impact parameter, careful *e*‐value selection while generating the MSA will reduce the noise sequences included in the MSA before tapping the distant sequences. Yang et al. [70] showed that a well‐chosen *e*‐value would impact the quality of MSA, then impact the success of protein 3D structure modeling. They also design a model, which could predict the optimal sequence distance information parameter (i.e., *e*‐value cutoff) used for constructing the MSA with the highest quality when given a Pfam family as input.

Second, we should emphasize that, from the ecological perspective, each biome is enriched for a specific set of phyla, which has been proved in previous research [79, 80, 81]. From the perspective of ecology, there are intricate but potentially important properties about protein structures that lead to their association with biomes, and the internal evolutionary and ecological drivers have shaped such properties: to adapt their biomes, functional genes from microbial species have to evolve so that the species could gain the advantage over other species in that specific niche, thus certain functional genes (or protein families) would highly likely to be enriched in a specific niche, though not exclusive to be present in such a niche.

## UNTARGETED AND TARGETED APPROACHES FOR PROTEIN STRUCTURE PREDICTION

Nowadays, many protein 3D structure prediction pipelines have been developed to utilize different metagenome sequences to supplement the homologous sequence (Table 1). With a rapidly increasing number of metagenome sequences, the metagenome utilization efficiency and marginal effect are critically important indicators of the effectiveness of metagenome data supplement for the protein structure prediction problem, methods that could improve the values of these two indicators could gain advantage for solving the problem.

**Table 1:** Approaches that could utilize metagenome data properties for better protein structure prediction​​​​​

Approach	Metagenome source	Number of biomes	Strategy	Source
HMM + Rosetta^a^	IMG database	Multiple biomes	Combined	[63]
HMM + C‐QUARK^b^	Ocean microbiome	Single biomes	Single	[64]
Alphafold^c^	Metagenome	Multiple biomes	Combined	[57]
DeepMSA + C‐I‐TASSER^d^	Mgnify	Multiple biomes	Combined	[70]
MetaSource + DeepMSA + C‐I‐TASSER^e^	Mgnify	Multiple biomes	Targeted	[70]

*Note*: Single strategy: using a single large biome as the protein source. Combined strategy: using a set of large biomes as protein sources. Targeted strategy: customized methods that select different biomes for different proteins.

^a^ Using IMG database [70, 82], models for 614 protein families were generated for unknown structures.

^b^ Using *Tara* Oceans data [80], proteins for 27 Pfam families were modeled with unsolved structures.

^c^ A deep learning algorithm, leveraging multisequence alignments were used for modeling protein structures.

^d^ Built on 4.25 billion microbiome sequences, 1044 Pfam families foldable by C‐I‐TASSER [70].

^e^ As targeted approach, MetaSource model was used to identify a set of biomes to supplement homologous sequence for specific Pfam families [70].

**Figure 5:**
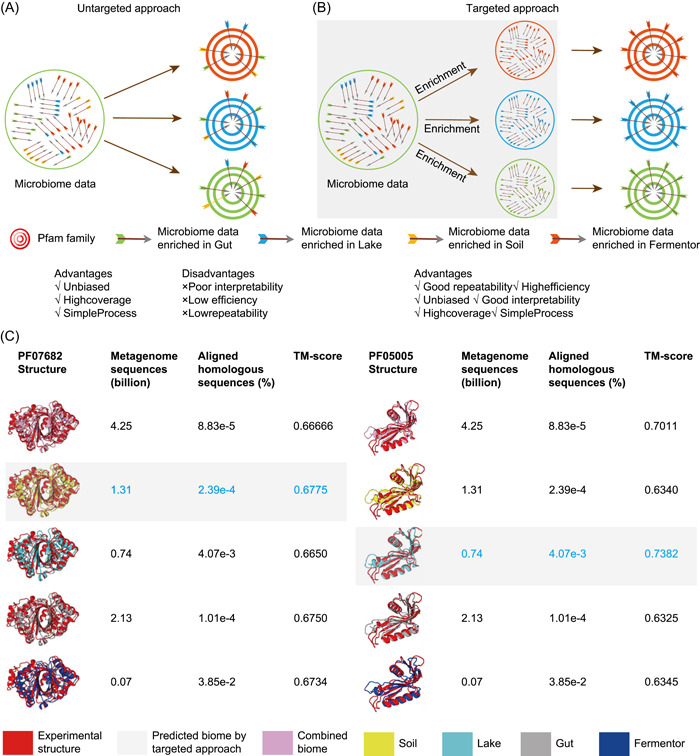
The targeted approach is essentially an enrichment approach. (A) Untargeted approach for the protein 3D structure prediction supplemented by metagenome. (B) Targeted approach for the protein 3D structure prediction supplemented by metagenome. (C) Case studies of modeling Pfam PF07682 and PF05005 with MSA from different biomes as the untargeted approach. For each biome, the number of metagenome sequences and the proportion of aligned homologous sequences in all the metagenome sequences was calculated. The correctness of 3D structure models was determined by comparing them to the known structure, which was quantified using the TM‐score method. The MetaSource is a targeted approach that was developed in a prior study [70]. The model labeled with gray background color is the source biome predicted by MetaSource. In blue type, the model with the highest TM‐score is displayed. 3D, three‐dimensional; MSA, multiple sequence alignment

The untargeted approach (Figure 5A), which is a method that finds homologous sequences from any source of the metagenome, does not have restrictions on the protein sequence search space. The entire process of an untargeted method lacks explanation and controllability since the association between metagenome data and the predicted proteins is not well known. Hence, the model training and metagenome search were mostly blind, and the source tracking of the most relevant biome datasets for individual protein targets was inefficient.

## TARGETED APPROACH COULD UTILIZE METAGENOME DATA PROPERTIES FOR BETTER PROTEIN STRUCTURE PREDICTION

While, compared to the untargeted approach, the targeted approach (Figure 5B) is a type of method that restricted the protein sequence search space. Instead of a blind search, a targeted strategy based on knowledge of the correlation between metagenome sequences might locate enough homologous sequences supplemented by the metagenome from specified biomes, which is favorable in terms of metagenome utilization efficiency and marginal effect.

For this purpose, the goal is simple: select a biome or a group of biomes for a given protein family, so that homologous sequences from this biome are much more enriched than those from other biomes. This MetaSource approach for guiding the source biome of metagenome data for supplementing protein structure prediction was born from this goal [70]: Based on the fact that different biomes enriched with different proteins, MetaSource trained with the Pfam families successfully modeled with a single biome. MetaSource was able to identify which biome would provide the most homologous sequences for protein, and the protein model supplemented by the metagenome from the predicted biome was validated with more accuracy than the protein model supplied by the metagenome from all biomes combined.

As a targeted approach, MetaSource not only predicts more precise protein structure but also outperforms untargeted approaches in terms of metagenome utilization efficiency. Evaluated by the data from previous research [70], MetaSource would be estimated with the metagenome utilization efficiency as 7810 homologous sequences per billion metagenome sequences, which is 50 times higher than the utilization rate using the IMG database (160 homologous sequences per billion metagenome sequences) [63] (Figure 3B). In other words, as a targeted approach, MetaSource can be used to decrease the time spent on the step of supplementing homologous sequences in protein structure prediction. This appears to be a critical area for a focused strategy since it has a direct impact on the efficiency of structure prediction.

For example, we have taken two Pfam examples from PF07682 and PF05005 with the known structure to evaluate the targeted approach and untargeted approach (Figure 5C) [70]. We also discovered that, even though the MSA from the combined biome contains more sequences than a single biome, the structural models from the combined biome are inferior to the MSA from a single biome (Soil or Lake), most likely owing to noise from irrelevant metagenome sequences (Figure 5C). As the targeted approach, MetaSource could forecast the right biome to model the protein 3D structure with the highest TM‐score, using much fewer metagenome sequences than the untargeted approach. The cause for this may be derived from the taxonomic profile found in the Pfam database: PF07682 and PF05005 are mainly composed of proteins from phylum Proteobacteria and Cyanobacteria, which dominate in Soil and Lake biomes, respectively [83, 84]. This result supports the advantage of the targeted approach: high coverage, high efficiency, and interpretability.

In summary, from the ecological and evolutionary perspectives, the metagenome utilization efficiency and marginal effect are crucial metrics for the effective prediction of protein structure from metagenome data, respectively. Metagenome utilization efficiency is highly data‐ and method‐dependent: on the data side, it is heavily dependent on the biomes from which the sequences are obtained; on the method side, an untargeted approach and targeted approach would lead to drastically different metagenome utilization efficiency. Furthermore, in many cases, the targeted approach would result in a more precise protein structure because of the less noise involved, as demonstrated by the comparison of the results based on two Pfam families.

## EXAMINATION OF THE BOUNDS FOR METAGENOME DATA NEEDED FOR PROTEIN STRUCTURE PREDICTION

Because template‐free methods rely on a high number of homologous sequences, it would be beneficial to anticipate the bound to represent all the proteins' reliable structures. Although the exact lower bound of metagenome sequences required for protein structure prediction is difficult to quantify, these bounds could be expected based on the same two key factors: metagenome utilization efficiency and marginal effect. Before estimating the bounds, we made a few simple assumptions: (1) from the current Pfam database [60], the number of proteins *N*(*P*_j_), the homologous sequences for a protein family Homo(*P*_j_), and the average homologous sequences for a protein AveHomo(*P*_j_) could be derived; (2) for current metagenome data (i.e., from IMG database [85], Mgnify database [86] and NCBI SRA database [87]), the number of biomes *N*(*B*_i_) might be determined; (3) based on previous work [63, 64, 70], metagenome utilization efficiency *UE*(*B*_i_, *P*_j_) for using metagenome data from a specific biome *B*_i_ for a specific protein *P*_j_, and the average metagenome utilization efficiency Ave(*UE*) could be calculated. (4) Based on previous work [63, 64, 70], marginal effect ME (*B*_i_, *P*_j_) for metagenome data from a specific biome *B*_i_ in supplementing homologous sequences for a specific protein *P*_j_ could be calculated.

Based on these assumptions, when an untargeted approach is used, a very rough estimation has shown that it would need an enormous amount of metagenome data without restriction on protein sequence search space. The total number of metagenome sequences that would be needed is:

(1) $$A\text{ve}\left(\text{UE}\right)=\sum_{1}^{N(p_{j})}\left(UE(B_{i},P_{j}))/N(P_{j}\right),$$

(2) $$\text{Sum}(\text{Seq})=N(P_{j})\times \text{AveHomo}(P_{j})\times /\text{Ave}(\text{UE}).$$

And based on current data statistics, AveHomo(*P*_j_) ∼ 3713, *N*(*P*_j_) is 19,179 based on Pfam 34.0 (http://pfam.xfam.org/). And Ave(*UE*) ∼ 100 per billion metagenome sequences. Thus, Sum(*Seq*) ∼ 7.12E12 is based on the most conservative estimation.

When the targeted approach is used, the bound of the number of homologous sequences could be largely reduced. For all proteins, the number of metagenome sequences is:

(3) $$\text{Sum}\left(\text{seq}\right)=\sum_{1}^{N(P_{j})}\left(\text{Homo}(P_{j})/\;\text{UE}(B_{i},P_{j})\right).$$

For this number, we can estimate the lower bound as 4.32E12. According to the data from previous research based on four representative biomes (Gut, Soil, Lake, Fermentor) [70], the average metagenome utilization efficiency (per billion metagenome sequence used for specific protein family) are Gut: 10, Soil: 248, Lake: 142, Fermentor: 320, respectively. And the average utilization efficiency is 180 per billion metagenome sequences, which is equivalent with (10(Gut) + 248(Soil) + 142(Lake) + 320(Fermentor))/4(number of biomes) (Equation 1).

**Figure 6:**
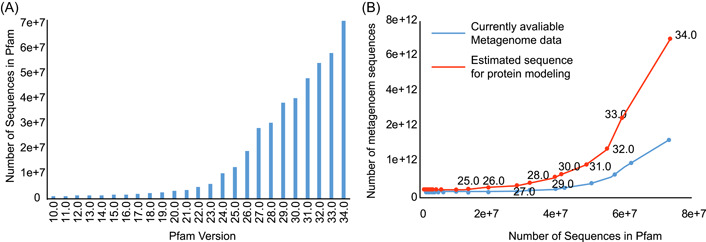
The relationships between the increasing number of proteins, and the increasing amount of metagenome sequences. (A) The number of sequences in Pfam under different versions. (B) The correlation between the number of metagenome sequences and the number of sequences in Pfam. Each node represents a Pfam release version

Taken together, we have created correlations between the rising number of proteins and the increasing number of metagenome sequences by combining our findings (Figure 6). With the increasing number of sequences in the Pfam database (Figure 6A), the gap between the number of protein sequences and the needed metagenome sequences is widening (Figure 6B). Given that the current Pfam database has 19,179 proteins, 7.12E12 metagenome sequences are estimated to predict all the protein structures but the current metagenome database only about 1.48E12 metagenome sequences (from three metagenome databases: IMG database [85], Mgnify database [86] and SRA database [87]). According to the data from previous research based on four representative biomes (Gut, Soil, Lake, Fermentor) [70], the targeted approach (lower bound was estimated as 4.32E12 by Equation 3) has a lower bound than the untargeted approach, owing to the targeted approach's greater average utilization efficiency (185 per billion metagenome sequences) than the untargeted approach (100 per billion metagenome sequences). It should be noted that this lower bound of the targeted approach is estimated based on using four representative biomes (Gut, Soil, Lake, Fermentor), yet it should already be clear that the lower bound of the targeted approach is small than that of the untargeted approach. Collectively, the targeted approach could substantially reduce the number of metagenome sequences required for this prediction purpose.

## DISCUSSIONS AND CONCLUSION

Protein 3D structures prediction supplemented by metagenome sequence is a very promising strategy for decoding the structure and function of the proteins, yet previous research has shown that such an approach is quite unstable. This study has revealed the data‐ and method‐dependent patterns behind this approach: The metagenome sequences from different biomes could contribute drastically different for a specific protein family, while the targeted approach could perform much better than the untargeted approach for protein family homologous sequence supplement.

From the ecological perspective, the problem of effective discovery of protein family homologous sequences is essentially a problem about ecological and evolutionary patterns of the proteins: to adapt their biomes, functional genes from microbial species have to evolve so that the species could gain the advantage over other species in that specific niche, thus certain functional genes (or protein families) are highly likely to be enriched in a specific niche, though not exclusive to be present in such a niche.

On the side of ecological modeling, the difficulty of finding homologous sequences in protein families is fundamentally an enrichment problem: from which biome or phyla we can most effectively excavate homologous sequences. And our assessment findings have already demonstrated that a targeted approach such as MetaSource could establish the link between microbes' habitats with homologous sequences, allowing us to deduce the sequential and structural aspects of functional genes from microorganisms' habitat information. This would prompt that the solved proteins would play important role in the predicted biomes and increase the interpretability of the whole targeted process.

On the side of evolutionary patterns, the targeted approach would anticipate the source biome for a protein to find enough evolutionary information (i.e., homologous sequences) to model its reliable structure. Different from the untargeted approach, which only provides the existing evolutionary information in the metagenome, the targeted approach would provide the guidance to find the evolutionary information that already exists in nature but has not been sequenced: If available metagenome cannot provide enough evolutionary information for proteins, the evolutionary information would be supplemented by sequencing the new metagenome samples from the predicted biome.

Furthermore, we estimated the lower bounds to predict the demands of metagenome sequences for predicting 3D structures for all the proteins in the Pfam database, and we discovered that current metagenome data could not meet the needs of metagenome sequences. On one hand, collecting more metagenome sequences could lead to more 3D structure prediction, while on the other hand, there is always a need to balance the prediction power and efficiency based on these huge number of metagenome sequences. For this proposal, the targeted approach would be the ideal alternative since it would boost metagenome utilization efficiency by reducing the search space and providing sufficient homologous information based on the knowledge of the ecological and evolutionary information in various biomes. In this regard, the focused strategy might significantly close the gap for this prediction purpose by enhancing the metagenome usage efficiency and guiding the subsequent homologous sequence supplement.

Collectively, the metagenome data utilization efficiency is profoundly improved by the targeted approach (exemplified by the MetaSource approach), demonstrating the targeted approach's enormous promise for protein structure prediction from metagenome sequences. When combined with another finding in this study that it is not necessarily true that more homologous sequence leads to better structure prediction, we deemed that the targeted approach is a win–win solution for protein structure prediction from metagenome sequences: it not only requires a drastically reduced number of sequences but also could improve prediction results for many protein families. On the other hand, the targeted approach has given us a wealth of knowledge regarding the ecological and evolutionary patterns of the proteins of interest.

## CONFLICTS OF INTEREST

The authors declare no conflicts of interest.

## AUTHOR CONTRIBUTIONS

Kang Ning conceived of and proposed the idea and designed the study. Pengshuo Yang and Kang Ning performed the review. All contributed to editing and proofreading the manuscript. All authors read and approved the final manuscript.

## Data Availability

The data that support the findings of this study are openly available at https://doi.org/10.1126/science.aah4043 [63], https://doi.org/10.1186/s13059-019-1823-z, [64], and https://doi.org/10.1073/pnas.2110828118, [70]. Supporting Information (tables, scripts, graphical abstract, slides, videos, Chinese translated version, and update materials) are available online DOI or GitHub https://github.com/iMetaScience/iMeta2022Ning.
